# Global Myeloma Research Clusters, Output, and Citations: A Bibliometric Mapping and Clustering Analysis

**DOI:** 10.1371/journal.pone.0116966

**Published:** 2015-01-28

**Authors:** Jens Peter Andersen, Martin Bøgsted, Karen Dybkær, Ulf-Henrik Mellqvist, Gareth J. Morgan, Hartmut Goldschmidt, Meletios A. Dimopoulos, Hermann Einsele, Jesús San Miguel, Antonio Palumbo, Pieter Sonneveld, Hans Erik Johnsen

**Affiliations:** 1 Medical Library, Aalborg University Hospital, Aalborg, Denmark; 2 Department of Haematology, Aalborg University Hospital, Aalborg, Denmark; 3 Department of Hematology Sahlgrenska Hospital, Gothenburg, Sweden; 4 The Institute of Cancer Research Haematology & Oncology, London, United Kingdom; 5 Nationales Centrum für Tumorerkrankungen, Heidelberg, Germany; 6 Department of Clinical Therapeutics, University of Athens, Alexandra General Hospital, Athens, Greece; 7 Department of Internal Medicine II, University of Würzburg, Würzburg, Germany; 8 Universidad de Navarra, Pamplona, Spain; 9 Department of Hematology, University of Turin, Turin, Italy; 10 Erasmus University Hospital, Department of Hematology, Rotterdam, The Netherlands; 11 European Myeloma Network Board, Aalborg, Denmark; 12 Clinical Cancer Research Center, Aalborg University Hospital, Aalborg, Denmark; 13 Department of Clinical Medicine, Aalborg University, Aalborg, Denmark; Katholieke Universiteit Leuven, BELGIUM

## Abstract

**Background:**

International collaborative research is a mechanism for improving the development of disease-specific therapies and for improving health at the population level. However, limited data are available to assess the trends in research output related to orphan diseases.

**Methods and Findings:**

We used bibliometric mapping and clustering methods to illustrate the level of fragmentation in myeloma research and the development of collaborative efforts. Publication data from Thomson Reuters Web of Science were retrieved for 2005–2009 and followed until 2013. We created a database of multiple myeloma publications, and we analysed impact and co-authorship density to identify scientific collaborations, developments, and international key players over time. The global annual publication volume for studies on multiple myeloma increased from 1,144 in 2005 to 1,628 in 2009, which represents a 43% increase. This increase is high compared to the 24% and 14% increases observed for lymphoma and leukaemia. The major proportion (>90% of publications) was from the US and EU over the study period. The output and impact in terms of citations, identified several successful groups with a large number of intra-cluster collaborations in the US and EU. The US-based myeloma clusters clearly stand out as the most productive and highly cited, and the European Myeloma Network members exhibited a doubling of collaborative publications from 2005 to 2009, still increasing up to 2013.

**Conclusion and Perspective:**

Multiple myeloma research output has increased substantially in the past decade. The fragmented European myeloma research activities based on national or regional groups are progressing, but they require a broad range of targeted research investments to improve multiple myeloma health care.

## Introduction

Multiple myeloma is an orphan malignant disorder of plasma cells. The incidence of multiple myeloma is 5–6 cases per 100,000 inhabitants. Knowledge of the essential pathogenic mechanisms and therapeutic possibilities has increased substantially over the last decade. In parallel, a broad range of novel technologies has resulted in new diagnostic, prognostic, and predictive procedures.

To exploit the clinical benefits of these recent developments, international collaborations have been organised through the European Myeloma Network (EMN), which was founded and legalised in 2003 by leading research groups. EMN is a Pan-European umbrella organisation that unites basic and clinical research groups. It provides a directory for these groups to pursue specific research and clinical goals together [1, 2].

The primary achievement of the EMN to date has been to introduce uniform criteria for diagnostic and prognostic assays in multiple myeloma. This has been accomplished through workshops focused on specific procedures related to biobanking, interphase FISH, multiparametric flow cytometry, and microarray technologies [3, 4]. The EMN has established two major working groups. The first is the “Biology Group”, which serves as an intermediary for the exchange of diagnostic tools and tumour samples [5–7]. The second group is the “Clinical Trial Group” which, in addition to publishing recommendations [8–12], has organised international collaborative clinical phase II and III trials supported by pharmaceutical companies.

The EMN is currently recognised internationally as a key promoter of interactions between researchers, companies, patient groups, and individuals working in different areas of the growing myeloma field. Therefore, the EMN provides a “golden opportunity” for continuing work on multiple myeloma and for preparing a strategy to support translation of basic discoveries into the clinic. In our aim to create an ideal future set-up for the EMN vision, we have used bibliometric mapping and cluster models to address questions related to the previous fragmentation of EU myeloma research. Our goals were to identify collaborative groups and to evaluate their outcomes and development over time. Finally, we relate our findings to other research fields with higher productivity, like lymphoma and leukaemia.

The findings of this study will allow us to restructure the EMN organisation and management, based on a strategy with focus on work that has high impact on individualized medicine. Implementation of individualized medicines has been limited but recent policy in EU has addressed central issues including biomarker validation, biobanking, clinical trials, data handling, public-private innovative medicines initiative and funding to personalized medicine. Initiatives, which future EMN work needs to take into consideration to speed up drug development and treatment strategies.

## Materials and Methods

### Analytic strategy

All bibliometric data were extracted from the Science Citation Index-Expanded (SCI-E) from Thomson Reuters Web of Science (WoS). The extraction was performed with topic queries for haematological malignancies, including multiple myeloma, lymphoma, and leukaemia. SCI-E is considered one of the largest citation databases in the world; it contains information on paper citation frequency and bibliographic metadata. Thus, it provides information that may not be retrieved from more specialised medical databases, such as PubMed MEDLINE or EMBASE. Citations to a paper can be used as a proxy for the impact a report has made on the research community, and therefore, the citation frequency can serve as an indicator of the importance, quality, or usefulness of the information [13].

Research papers on the disease areas of interest published during the years 2005–09 were identified as shown in S1 Table. This list was compared to results from similar queries performed in PubMed MEDLINE with medical subject headings (MeSH).

In the scientific literature, there are a variety of manuscript types, but in principle, a count of publications should only include peer-reviewed research papers. Articles and reviews are normally peer-reviewed, and these were included in our counts. Letters constitute a heterogeneous category. They include peer-reviewed short communications, but also un-refereed correspondences. It was not algorithmically possible to distinguish between the two types of letters. However, we considered it more important to include the peer-reviewed letters than to exclude non-peer reviewed letters; therefore, we decided to include all letters.

To compare the geographical affiliation of collaborating authors, we retrieved the country-element of the addresses registered for the authors of each paper. We defined European countries as the 28 countries of the EU plus Switzerland, Norway, Iceland, Macedonia, and Turkey, based on their central location in the EU and/or their candidacy to become an EU member state. We compared papers from the EU to those from the US and those from the Asian continent, which included Russia. In most cases, these three groups explained close to 100% of the addresses identified.

We used both the names and addresses of authors from the SCI-E-data. These data were given in the journal, typically supplied by the authors. Nevertheless, these data may have contained spelling errors, due to a lack of standards or differing standards between journals. We only used the country information of addresses; therefore, this was a minor issue. All country information was easily standardised.

Variations and ambiguity in author names were resolved in some systems by canonical or authorised author names. Although WoS used the so-called ResearcherID, this system was not fully incorporated in the entire database, nor was the more recent OrcID system available for cases of retrieval. Thus, cases of two or more authors that used the same name or cases with different spellings of the same name remained problematic. Some attempts have been made to resolve these issues [14–16], primarily by using co-author-based clustering techniques. In the present study we used initial clusters to identify spelling variations of author names combined with manual searching for the more prominent author names. The disambiguation was performed by one of the authors with no prior knowledge of the field and subsequently evaluated by the other authors with expert knowledge of this field. While no clustering technique or manual approach will ever produce a completely accurate picture of authorships, our expert evaluation suggests an acceptable margin of error.

### Bibliometric method and mapping

The bibliometric method was based on identifying particularly strong collaborations between authors, based on the number of co-authored papers [17–19]. Traditionally, maps of author collaborations are created by first applying similarity matrices, based on the number of co-authored papers, to construct clusters of authors that work on similar topics [19]. Then, a mapping technique was used to illustrate the clusters. We used Pajek64, version 3.15 (http://pajek.imfm.si/doku.php) for co-author network analysis. For clustering, the Louvain community clustering method [20] was used, with resolution parameter 1.0 and multi-level coarsening and refinement. The choice of resolution parameter was based on a combined comparison with hierarchical agglomerative clustering (not shown) and expert prior knowledge about the multiple myeloma field. As the default resolution parameter of 1.0 provided meaningful clusters in the multiple myeloma dataset, the same value was also used for the other datasets. For mapping, the Kamada-Kawai layout was used with internal cluster optimisation, as this layout provided the most clearly visible structures. The clustering was performed by means of all authors with at least 20 publications in the entire time frame and all edges were included in all calculations. For the visual layout however, edges with values below 10 were removed to provide a clearer image. This has the consequence that some inter-cluster links are not shown. The maps were also created with VOSviewer version 1.5.5 for comparison, resulting in comparable clusters and maps (data not shown).

Clusters were evaluated using two measures of cluster density: clustering coefficients for immediate neighbours (*CC1*) and closeness centrality (*C_close_*). While both measures express a variant of density, they differ somewhat in their interpretation. The *CC1* indicates for each member of a network how many other members are in the neighbourhood compared to the total potential neighbours [21], while the *C_close_* indicates the average (inverse) shortest distance from any member of a network to all other members [22]. As both indicators work on entire graphs, clusters were extracted as subgraphs before calculating these measures in Pajek.

### Key indicators of productivity and impact

The productivity and impact of research groups were assessed by numbers of publications and citations.

1) For publication counts, we measured full and fractional counts [23] to show actual differences and relative productivity. Comparing publications among clusters depended on:

- Cluster publication counts, *N_c_*, or the full count, was defined as the total number of unique papers per cluster, where at least one author from the cluster was listed as an author, regardless of whether it was first, last, or any other place in the author list.
- Fractional cluster author counts was defined as $p_{fc}=\sum_{i=1}^{N}n_{i}/a_{i}$, where *n_i_* was the number of authors from the respective cluster that appeared on a paper *i, a_i_* was the total number of authors on paper *i*, and *N* was the total number of papers that listed at least one author from the cluster.
- Intra-cluster collaboration was defined as the ratio *p_fc_*/*N_c_*, or the proportion of co-authorships within a cluster by authors from that cluster.

2) We used the total citation counts at the date of collection, and standardised these according to year of publication. While some impact indicators use field standardisation to compensate for differing citation densities (e.g. [24]), the data used in this paper is from four closely related subjects, each of which is defined on a more specific level than the WoS subject categories otherwise available for field standardisation. Thus we consider a publication year standardisation sufficient. Standardisation can incorporate several elements, such as log-normalisation, omission of uncited papers or z-scores, however; to provide the most transparent normalisation, we chose to divide the citations for any paper *i*, *c_i_*, by the mean citations to papers published the same year and for the same disease category. We denote the normalised citations for paper *i* as *s_i_* and the mean normalised citation score of all papers for a given group as *µ_s_*. These mean normalised citation scores should be interpreted with care, especially for small groups, due to the highly skewed nature of citations [25]. We counted the actual citations that referred to papers instead of, e.g., the impact factor of the journals in which the papers were published. This approach achieved a more direct measure of the impact of the individual papers. It was previously shown that citations to papers in any given journal are highly skewed [25]. This tendency towards skewness is observed in most bibliometric variables, such as journals, authors, universities, countries etc. Therefore, the journal impact factor is rarely descriptive of a single paper. It is important to note, that while the citation normalisation approach described above suffers from the same vulnerability as the journal impact factor, we do not use it to describe single papers but only for aggregates such as clusters of author, countries and research groups. To further compensate for the skewness of citations, we also use a top-decile indicator, which we denote as *PP_top10_*, inspired by the similar indicator used in the CWTS Leiden ranking system [24]. For each disease category we find the normalised citation score of the paper ranking at the top decile limit, *s_D10_*. All papers in this disease category with si above this score are considered top papers, and the *PP_top10_* indicator can subsequently be calculated for any set of papers *A* as *PP_top_*_10_ = |*A* ∩ *D*10|/|*A*|, where |.| denotes the cardinality of a given set and D10 the set of top decile papers. The expected score would be 0.1, with values above indicating higher than expected performance.

A plethora of other metrics exists. However, we believe for comparing the impact of research groups in a given time period for so closely related research areas, the chosen indicators are the best suited. In particular when the mean- and percentile-based indicators are used together, as the latter can compensate for potential skewness of the former [24, 26–28].

### Data validation and follow up

A comparison between SCI-E and PubMed MEDLINE databases, based on medical subject headings (MeSH) for publications during the years 2005–09 is given in S1 Table. A large number of the publications were meeting abstracts registered only in the SCI-E. However, the number of scientific reports, reviews, and letters registered seemed to increase steadily over time for multiple myeloma, lymphoma, and leukaemia (S2 Table). For multiple myeloma the relative increase over time is 42% while it is 22% and 14% respectively for the other diseases, showing a far greater relative growth in multiple myeloma research.

A follow-up was performed for multiple myeloma specifically, in order to track productivity and impact over a longer period of time. The dataset was thus broadened to all multiple myeloma papers from 2003–2013. This dataset is analysed separately, as the other diseases were not analysed over the same period of time, and impact analyses for recent years are somewhat less stable.

## Results

### Cluster identification and research output

**Fig 1:**
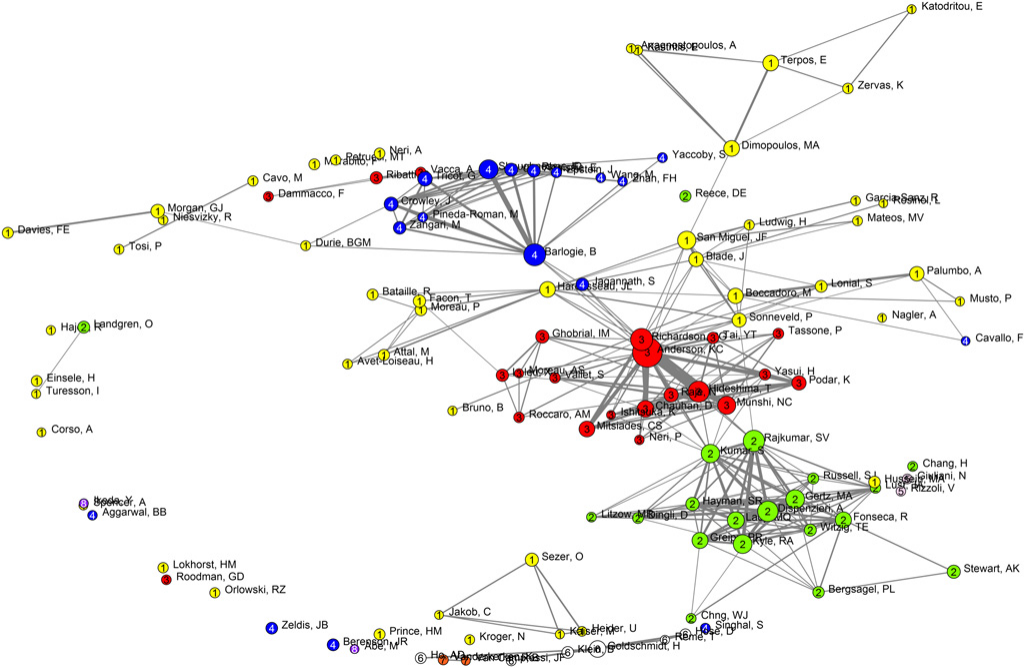
Multiple myeloma mapping and clustering of authors with >20 publications. These network maps illustrate the collaboration networks of all authors in the datasets. Each vertex in the map illustrates an author, the size of the vertex indicates the number of papers published by this author, on the respective subjects in the given time period. The thickness of lines between authors indicates the degree of collaboration between them and the colours indicate which authors are associated with the same cluster. EU members form the clusters my1, my6, my5, and my7. My1 is the yellow cluster spread over the majority of the map, while my6 is shown as a white cluster towards the bottom. My5 and my7 are two-person clusters. The majority of EMN members are present in my1, with the exception of H Goldschmidt from GMMG, Germany, in cluster my6. The three most highly cited clusters from the US, my2, my3, and my4 include the red cluster, which includes KC Anderson from Dana-Farber Cancer Institute (DFCI), Boston, MA, the blue cluster, which includes B Barlogie from the University of Arkansas Medical Sciences (UAMS), Little Rock, AR, and the green cluster, which includes A Dispenzieri and SV Rajkumar from the Mayo Clinic in Rochester, MN, Phoenix, AZ, and Jacksonville, FL.

Collaborative clusters were successfully identified using the Louvain clustering algorithm. Bibliometric co-author maps were constructed for multiple myeloma (Fig. 1), lymphoma (S1 Fig.), and leukaemia (S2 Fig.). We only included authors with *>* 20 publications during the time period indicated. Each map illustrates the collaborating networks; the vertices (or circles) illustrate one author, the circle size indicates the number of publications in the given time period, the thickness of edges between vertices the degree of collaboration, and common colours show clusters of associated authors. Numbers inside the vertices are IDs of clusters, which in the following tables are used in combination with disease codes (my = myeloma, le = leukemia etc) so that my1 is cluster 1 in the multiple myeloma map.

The clusters were analysed for productivity and other descriptive data (Table 1, S3 and S4 Table). Productivity was reflected by the total number of publications in the cluster (*N_c_*), and the fractional count of publications per cluster (*p_fc_*). It should be noted that the clusters did not contain all papers in the datasets, as they were generated from authors with at least 20 publications in the time period. The publication rates differed for multiple myeloma, lymphoma, and leukaemia, with medians of 211 (range 27–794), 341 (range 20–1164), and 328 (range 19–2649), respectively. While the omission of authors with less than 20 papers leads to a reduction in the total data, it was necessary to use a threshold in order to allow interpretation of the maps. The results should thus be seen as representative of the most productive researchers rather than the entirety of multiple myeloma research.

**Table 1:** Descriptions of multiple myeloma research clusters ranked by publication counts.

**Cluster ID**	***N_c_***	***p_fc_***	***p_fc_/Nc***	***µ_s_***	***PP_top10_***	***C_coeff_***	***C_close_***	**EU**	**US**	**Asia**
my1	794	227.66	0.287	1.48	0.16	0.789	0.384	74.10%	16.10%	1.10%
my2	387	159.32	0.412	1.93	0.23	0.912	0.249	27.60%	51.90%	4.10%
my3	364	147.03	0.404	1.9	0.24	0.851	0.362	44.50%	45.60%	5.40%
my4	327	102.06	0.312	2.06	0.26	0.847	0.446	28.10%	59.30%	4.80%
my6	95	19.4	0.204	1.45	0.11	1	0	83.90%	6.60%	0.50%
my8	40	4.89	0.122	0.67	0.05	0	0	2.40%	0.00%	95.10%
my5	27	7.41	0.275	1.2	0.15	0	0	88.20%	8.80%	2.90%
my7	27	6.19	0.229	0.99	0.11	0	0	80.30%	16.40%	3.30%

Descriptive data for all clusters: *N_c_* = total publication count; *p_fc_* = fractional publication count; *p_fc_*/*N_c_* = ratio between *p_fc_* and *N_c_*, indicating degree of intra-cluster collaboration; *µ_s_* = mean standardised citation score; *PP_top10_* = proportion of publications from the top decile; *C_coeff_* = 1-neighbourhood cluster coefficient; *C_close_* = mean closeness centrality for the cluster; EU, US, Asia = fraction of addresses on papers from the respective areas.

The intercontinental and worldwide collaborations for each cluster were graded by calculating the distribution of EU, US, or Asian partners. The most specific collaboration-indicator was the ratio between the fractional and the full publication counts (*p_fc_/N_c_*). The closer this number was to 1, the greater the intra-cluster collaboration; i.e., a larger number of authors on each publication belonged to the same cluster. Table 1, S3 and S4 Table show that this indicator (*p_fc_/N_c_*) for multiple myeloma, lymphoma, and leukaemia had medians of 0.281 (range 0.122–0.412), 0.256 (range 0.117–1), and 0.258 (range 0.189–1), respectively. The medians for each disease are comparable, indicating similar strengths in collaborations regardless of disease, which should be seen in spite of disease-specific differences, such as the relatively lower incidence rate of multiple myeloma. We thus compared the multiple myeloma clusters to acute myeloid leukaemia (AML) research, another bone marrow disease with the same incidence (S5 Table). We found that the collaboration indicator (*p_fc_/N_c_*), median 0.195 (range 0.124–0.325), was somewhat lower for AML, which supports the notion that multiple myeloma research has closer intra-cluster collaboration than could be expected.

### Cluster impact by citations

The impact of publications was indicated by the mean standardised citation score (*µ_s_*) as shown in Table 1, S3 and S4 Table. The *µ_s_* values for multiple myeloma, lymphoma, and leukaemia were 1.465 (range 0.67–2.06), 1.42 (range 0–2.28), and 1.075 (range 0–2.49), respectively. These results indicated that the multiple myeloma clusters had a high level of impact. Again, we compared multiple myeloma research with acute myeloid leukaemia research, and found a median *µ_s_* = 1.47 (range 0.23–2.17), with highest values for the US clusters (S5 Table). Comparably, among the multiple myeloma clusters, the US also had higher *µ_s_* values (2.06, 1.93, and 1.90) than the identified EU clusters (1.48, 1.45, 1.20 and 0.99) (Table 1). In other words, the high level of research impact observed in the multiple myeloma clusters depended on the citations from the US, anchored in clusters my2, my3, and my4. Note that my3 has almost equal EU and US contributions, but is classified as a US cluster here, as the US is a single contry responsible for almost half of the addresses present in the cluster, while the EU represents several countries

### Key players in multiple myeloma research

**Fig 2:**
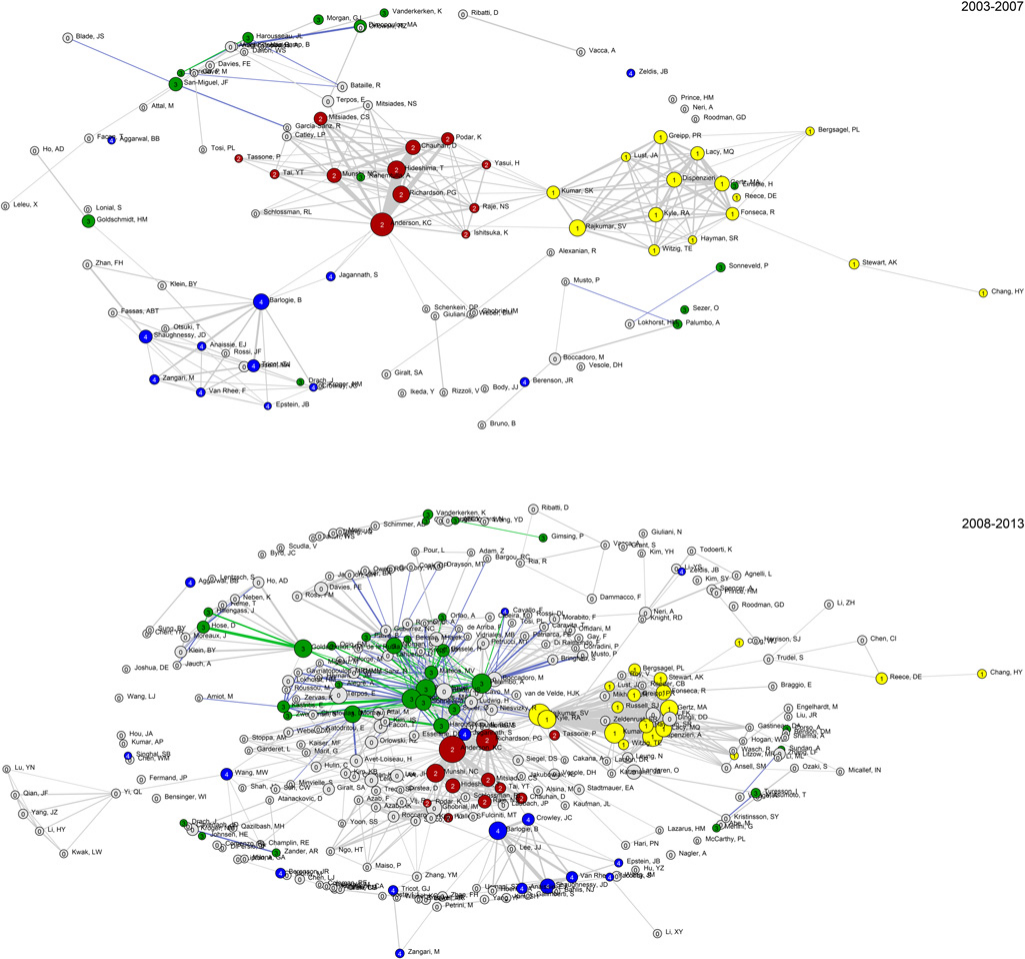
Co-author cluster changes from 2003–2007 to 2008–2013. This network diagram illustrates the change in collaborative research over the periods shown; only authors with more than 20 papers in each period are included. Green circles represent EMN members, while the other colours represent the three major US clusters (my2: yellow, my3: red, my4: blue). The green edges represent inter-EMN collaboration while blue edges represent collaboration between EMN and non-EMN authors.

An overview of the multiple myeloma-related cluster scores is displayed in Table 1, and the corresponding co-author map is shown in Fig. 1. Most importantly, compared to the US-based clusters, the edges inside EU clusters are much smaller, indicating a lower intra-cluster collaboration rate during 2005–2009 (Table 1 and Fig. 1). However, during the interval from 2003 to 2013, the EU clusters move toward each other (Fig. 2, map for 2008–2013); this movement was most likely a consequence of the EMN collaboration initiated in 2003. This observation is further discussed in the time dependent analysis of multiple myeloma research reported below.

Of interest, among the three US clusters, my2 and my3 are institutional members of the Multiple Myeloma Research Consortium (MMRC), founded in 2004. This consortium brought together an extraordinary group of US myeloma researchers and world-renowned institutions to speed up translational and clinical developments in multiple myeloma. During 2005–2009, the three US clusters were well defined, with relatively abundant intra-cluster collaborations (Table 1 and Fig. 1).

### Key players in lymphoma and leukaemia research

The lymphoma related cluster scores are given in S3 Table. The highest production and impact were observed in clusters ly8, ly11, ly6, ly2, and ly1. Researchers from the EU Lymphoma Institute (ELI) were identified in ly5 and ly7.

The leukaemia related cluster scores are given in S4 Table. One of the three most highly cited clusters (le4), mainly consists of EU researchers, while the other two are mostly US-based. Other large clusters from the EU are le7, le16, le12, le3, le8, and le14.

### Time dependent development for the multiple myeloma clusters

**Fig 3:**
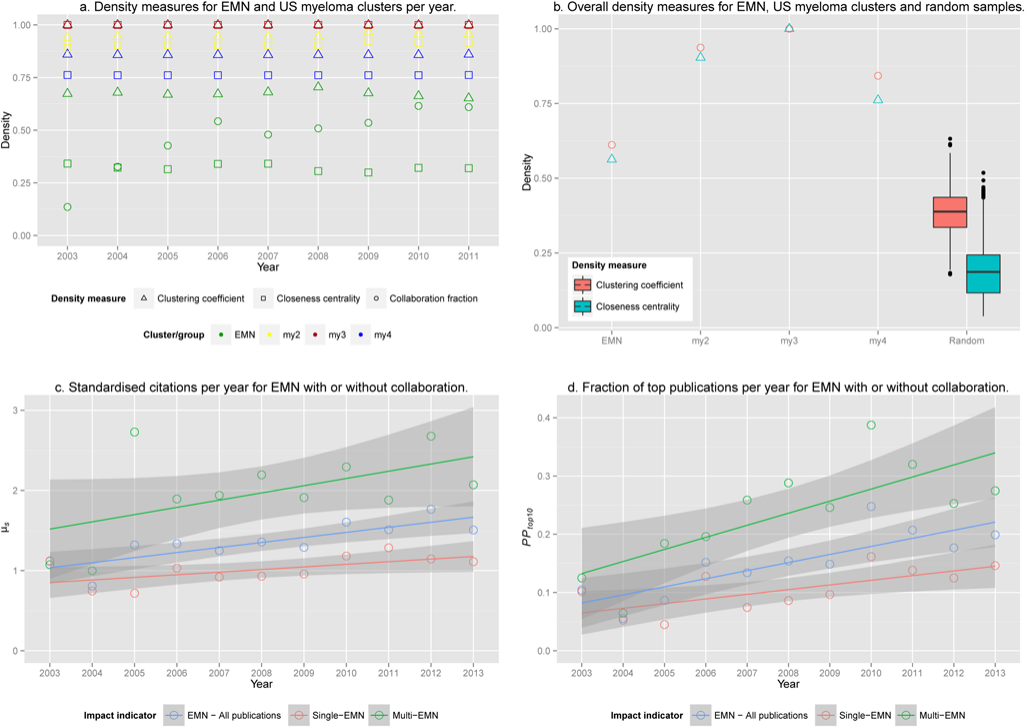
Time-dependent progress for the multiple myeloma networks. A: The two cluster density measures are shown for three year intervals (each data point is calculated from the displayed year and two years ahead to allow networks to form). The collaboration fraction is calculated as the number of papers with more than one author from the EMN, divided by all papers with at least one author from the EMN. B: the cluster density measures for the entire period are compared to 1,000 randomly drawn groups of the same size as the EMN group. C: The *µ_s_* scores per year for the EMN group, when considering all publications (blue) versus those with only one EMN member (red) and at least two EMN members as authors (green). The shaded areas indicate the.95 confidence intervals. D: Similar to C, but using the *PP_top10_* indicator.

To analyse the development of the EMN and the three major US-clusters (my2, my3, and my4) over time, the multiple myeloma dataset was extended from 2003 to 2013, using the same approach as that used for the main dataset. The cluster density indicators CC1 and closeness centrality are reported in Fig. 3a, along with a collaboration fraction indicator. This final indicator was only calculated for the EMN members and is the ratio between number of publications co-authored by two or more EMN members and those with only one author from the EMN. The three US clusters are very dense regardless of which indicator is used, while the EMN members do not collaborate as densely, in particular when looking at the closeness centrality. However, when the networks are plotted for two time periods (2003–2007 and 2008–2013, Fig. 2) the number of edges between EMN members indicates a tighter collaboration, while the weights of the edges indicates that these collaborations do not occur as frequently as for the US clusters. This observation is strengthened by the growing collaboration fraction (Fig. 3a) while the cluster densities are stagnant. We interpret this as growing EMN collaboration, but less systematically than in the US clusters, so that the probability of any two EMN members collaborating grows over time. However, all EMN members will rarely collaborate on the same publication, and they will be more likely to swap collaborators from publication to publication. The growth in collaboration may have been a consequence of the EMN activities. As it is apparent from Fig. 3a the cluster densities for the EMN members are lower than for the three clusters. It is, however, obvious that they should be lower, as the clusters are chosen to be dense clusters, while the EMN group is not a cluster chosen by a density criterion, but a given group of authors which do not necessarily all collaborate. We therefore compared the EMN group and US clusters to a set of randomly selected groups of authors. The random distribution was generated from the complete time period, as were the cluster measures for the other aggregates in Fig. 3b. Thousand random draws of 46 authors with minimum 20 papers. Theoretically, the EMN group of 46 authors can be drawn from this random sample, which is why we assessed the probability of getting a group with more extreme cluster behaviour than the EMN group by random selection. These two-tailed *p*-values were found from the quantile rank of EMN scores in the random distributions. For the closeness centrality, EMN scores were above any recorded values (*p* = 0.000), and for the clustering coefficient, the EMN scores were in the upper 0.2% (*p* = 0.004). This provides evidence that the EMN group collaborates more closely than what could be expected if they had just been a randomly selected group of authors.

In Fig. 3c and 3d we show the development in citation impact over time for the EMN-authored publications. Whether using the mean normalised citation rate, *µ_s_*, or the top-10% fraction, it is clear that publications where EMN members collaborate (Multi-EMN) have a higher citation impact than those where only one of the authors is from the EMN (Single-EMN).

**Fig 4:**
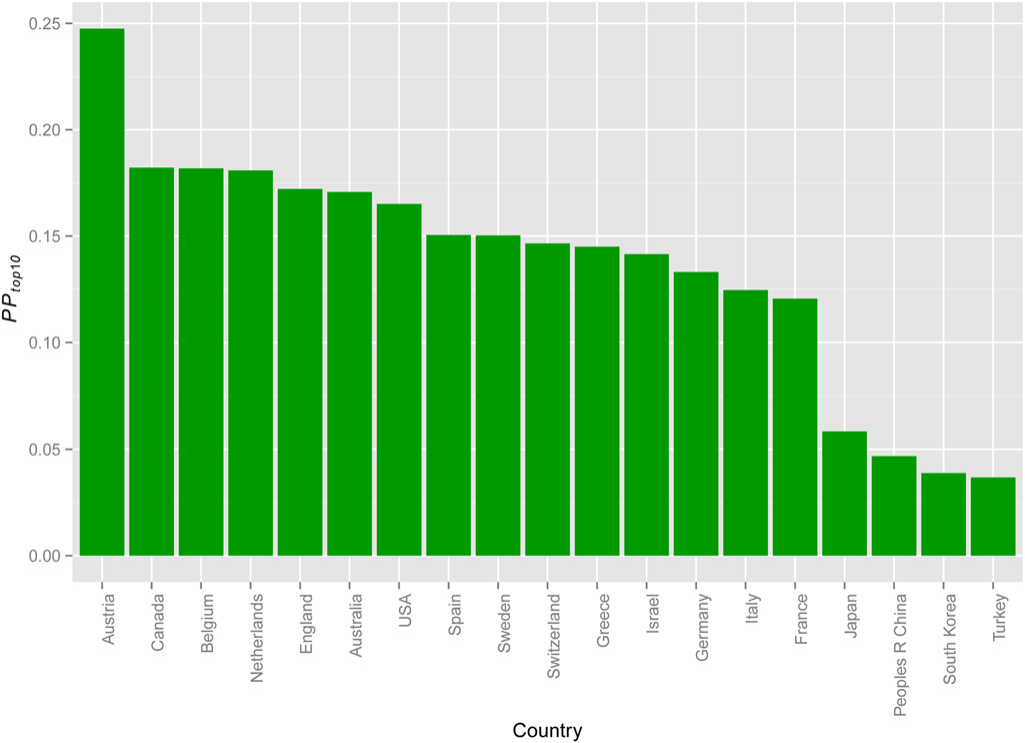
Proportion of top decile cited papers per country. The proportion of top decile cited papers per country, indicated by green bars. Countries are ordered by *PP_top10_*.

We analysed the impact of research, according to nationality, by comparing the *PP_top10_* scores per country for multiple myeloma research (Fig. 4). The results indicated that the research impact in most European countries matches the US level.

## Discussion

Before the turn of the millennium, basic and applied research in the field of multiple myeloma made slow progress. This situation has changed, due to a broadening interest in basic research, evolving drug designs, and novel therapies in both the EU and US. Until recently, most EU research activities lacked coordination; thus, unlike in the US, very few basic research endeavours have resulted in major clinical developments or breakthroughs. It was evident that major benefits could be gained from coordination, integration, and harmonization of activities, as suggested by the EU Framework Programmes (FP). Originally, the FP initiated the EMN and united 12 leading European institutions and national cooperative clinical trial groups, including the Haemato Oncology Foundation for Adults in the Netherlands (HOVON; The Netherlands, Belgium), Central Myeloma Study Group (CEMSG; Austria), Deutsche Studiengruppe Multiples Myelom (DSMM; Germany), German Speaking Myeloma Multicenter Group (GMMG; Germany), Nordic Myeloma Study Group (NMSG; Sweden, Denmark, Norway, Finland, and Iceland), Gruppo Italiano Malattie EMatologiche dell′Adulto (GIMEMA; Italy), Medical Research Council (MRC; UK), and Programa para el Estudio de la Terapéutica en Hemopatía Maligna (PETHEMA; Spain). In addition, the network included groups from Greece, the Czech Republic, Israel, and Turkey. Unfortunately, this network was unsuccessful in applying for Network of Excellence status and procuring support from the FP6^th^. Although most partners have established a range of EMN activities, the question remains as to whether the EMN collaborative outcome and impact over the last decade have paralleled the improvements in treatment outcome for patients.

The present report aimed to identify scientific collaborations, output, impact, international key players, and research developments over time by mapping the international research networks for multiple myeloma. The analysis of publication productivity and impact identified several successful groups with a high level of intra-cluster collaboration in the EU and US. However, US-based research clearly stands out as the most highly cited, even though EU research groups have considerable impact and have shown increased activity over time. These findings may guide us in restructuring the EMN organisation and infrastructure to implement a scientific strategy that focuses on specific areas that have potential impact on translational research, drug development and patient care.

The new era of ‘omics’ information will allow implementation of individual treatment strategies. A range of recent policy initiatives in EU address this issue and accelerate international projects toward personalized medicine. It is increasingly important to identify individuals who are unlikely to benefit from current treatment, or are at high risk to develop long-term side effects and therefore may benefit from novel or selected treatment regimens. This approach, personalized or precision medicine, is the key to reduction of the burdens of healthcare. Not only will it improve clinical outcomes, but it will ensure an efficient use of healthcare resources when fully developed. Personalized medicine includes diagnostics, biomarkers and drug development making a more complete understanding of the underlying disease mechanisms a prerequisite. Initiatives that facilitate the adoption of personalized medicine are steadily progressing in Europe, including European Medicines Agency (EMA) working parties, Innovative Medicines Initiatives (IMI) and Horizon2020. Personalized medicine will not succeed without grant support to health care professionals with interest in rare diseases, to ensure benefit for health cost-effectiveness. EMN represents such an organisation, in urgent need for EU support.

The main infrastructure of the EMN includes the Secretariat, placed in Denmark (Aalborg/NMSG), the EMN Data and Statistical Centre, placed in Italy (Torino/GIMEMA) and the Netherlands (Rotterdam/HOVON), the EMN Sponsor Offices in Denmark and the Netherlands, the National and Regional Clinical Research Units, and the Network of European Myeloma Biobanks and Laboratories. The EMN has been approved by the European Haematology Association (EHA) as an official scientific working group. The EMN also provides clinical recommendations and organises major clinical phase II-III trials to be carried out across the EU, with financial support from pharmaceutical companies. From unrestricted grants to the organization, the EMN has supported and facilitated the advent of the Myeloma Stem Cell Network (MSCNET), the translational project on Resistance and Targeted Therapy (RESTART), the Clinical Trial Network (TALISMMAN), and the MyelomA Genetics International Consortium (MAGIC).

The results from this analysis are retrospective and indicative in nature. However, the most significant observation was the intercontinental difference between the EU and US in outcome and impact. We observed indications that the major performance group in multiple myeloma research, my3, includes awardees of the 2.7 million USD SPORE Grant [29] and collaborations with researchers from one of the other US-groups, my4. The other US-based research group, my2, is supported by a 3 million USD grant from the US NCI [30]. The NCI investment in multiple myeloma research has increased steadily, from $41.5 million in 2008 to $61.3 million in 2012. In addition to that investment, the NCI invested $5.6 million to support multiple myeloma research in 2009 and 2010 with funding from the American Recovery and Reinvestment Act [31].

There are no differences between the EU and US in the incidence and prevalence of multiple myeloma. It has been estimated that more than 20,000 individuals will be diagnosed with multiple myeloma in the US and in the EU during 2014, and more than 10,000 patients will die annually as a result of resistant disease. Although we cannot draw any direct conclusions from our analyses, it may not be surprising that well-funded, collaborative, focused research projects produce intra-cluster collaborative progress and high-impact research papers. The EU investment in multiple myeloma research is unknown, but the EMN partnership has estimated that the investment is far below the US level.

We conclude that the fragmented European multiple myeloma research activities performed by national or regional groups are progressing towards international collaborations. Therefore, our findings can provide the basis for an updated EMN strategy for achieving the content and output expected from future multiple myeloma research. This strategy should be decided by the EMN board and leading partners from the Myeloma Biology and Clinical Trial Groups. A clearly defined “top down” strategy for EMN will give all members a precise description of the achievements needed to apply successfully for Horizon 2020 grant support.

## Supporting Information

S1 Fig Lymphoma mapping and clustering of authors with > 20 publications. This network maps illustrate the collaboration networks of all authors in the datasets. Each vertex on the map illustrates an author, the size of the vertex indicates the number of papers published by this author, on the respective subjects (S3 Table), in the given time period. The thickness of lines between authors indicates the degree of collaboration between them and the colours indicate which authors are associated with the same cluster.
(TIF)

S2 Fig Leukaemia mapping and clustering of authors with > 20 publications. This network maps illustrate the collaboration networks of all authors in the datasets. Each vertex on the map illustrates an author, the size of the vertex indicates the number of papers published by this author, on the respective subjects (S4 Table), in the given time period. The thickness of lines between authors indicates the degree of collaboration between them and the colours indicate which authors are associated with the same cluster.
(TIF)

S3 Fig Acute myeloid leukaemia mapping and clustering of authors with 10 or more papers. This network maps illustrate the collaboration networks of all authors in the datasets. Each vertex on the map illustrates an author, the size of the vertex indicates the number of papers published by this author, on the respective subjects (S5 Table), in the given time period. The thickness of lines between authors indicates the degree of collaboration between them and the colours indicate which authors are associated with the same cluster.
(TIF)

S1 Table Search results compared between Web of Science and PubMed. Queries for multiple myeloma, lymphoma, and leukaemia in Science Citation Index-Expanded (SCI-E) and PubMed MEDLINE, delimited to publication years = 2005–2009. The refined SCI-E query contains only articles, reviews, and letters.
(PDF)

S2 Table Counts of papers over time. Publication counts in SCI-E distributed by publication year.
(PDF)

S3 Table Descriptions of lymphoma research clusters ranked by publication counts. Descriptive data for all clusters: *N_c_* = total publication count; *p_fc_* = fractional publication count; *p_fc_*/*N_c_* = ratio between *p_fc_* and *N_c_*, indicating degree of intra-cluster collaboration; *µ_s_* = mean standardised citation score; *PP_top10_* = proportion of publications from the top decile; *C_coeff_* = 1-neighbourhood cluster coefficient; *C_close_* = mean closeness centrality for the cluster; EU, US, Asia = fraction of addresses on papers from the respective areas.
(PDF)

S4 Table Descriptions of leukaemia clusters ranked by publication counts. Descriptive data for all clusters: *N_c_* = total publication count; *p_fc_* = fractional publication count; *p_fc_*/*N_c_* = ratio between *p_fc_* and *N_c_*, indicating degree of intra-cluster collaboration; *µ_s_* = mean standardised citation score; *PP_top10_* = proportion of publications from the top decile; *C_coeff_* = 1-neighbourhood cluster coefficient; *C_close_* = mean closeness centrality for the cluster; EU, US, Asia = fraction of addresses on papers from the respective areas.
(PDF)

S5 Table Descriptions of acute myeloid leukaemia clusters ranked by publication counts. Descriptive data for all clusters: *N_c_* = total publication count; *p_fc_* = fractional publication count; *p_fc_*/*N_c_* = ratio between *p_fc_* and *N_c_*, indicating degree of intra-cluster collaboration; *µ_s_* = mean standardised citation score; *PP_top10_* = proportion of publications from the top decile; *C_coeff_* = 1-neighbourhood cluster coefficient; *C_close_* = mean closeness centrality for the cluster; EU, US, Asia = fraction of addresses on papers from the respective areas.
(PDF)
