# Supplementary figures and images for: Global Myeloma Research Clusters, Output, and Citations: A Bibliometric Mapping and Clustering Analysis
Source: PLoS One. 2015 Jan 28;10(1):e0116966. doi: 10.1371/journal.pone.0116966 (PMC4309532; doi:10.1371/journal.pone.0116966)

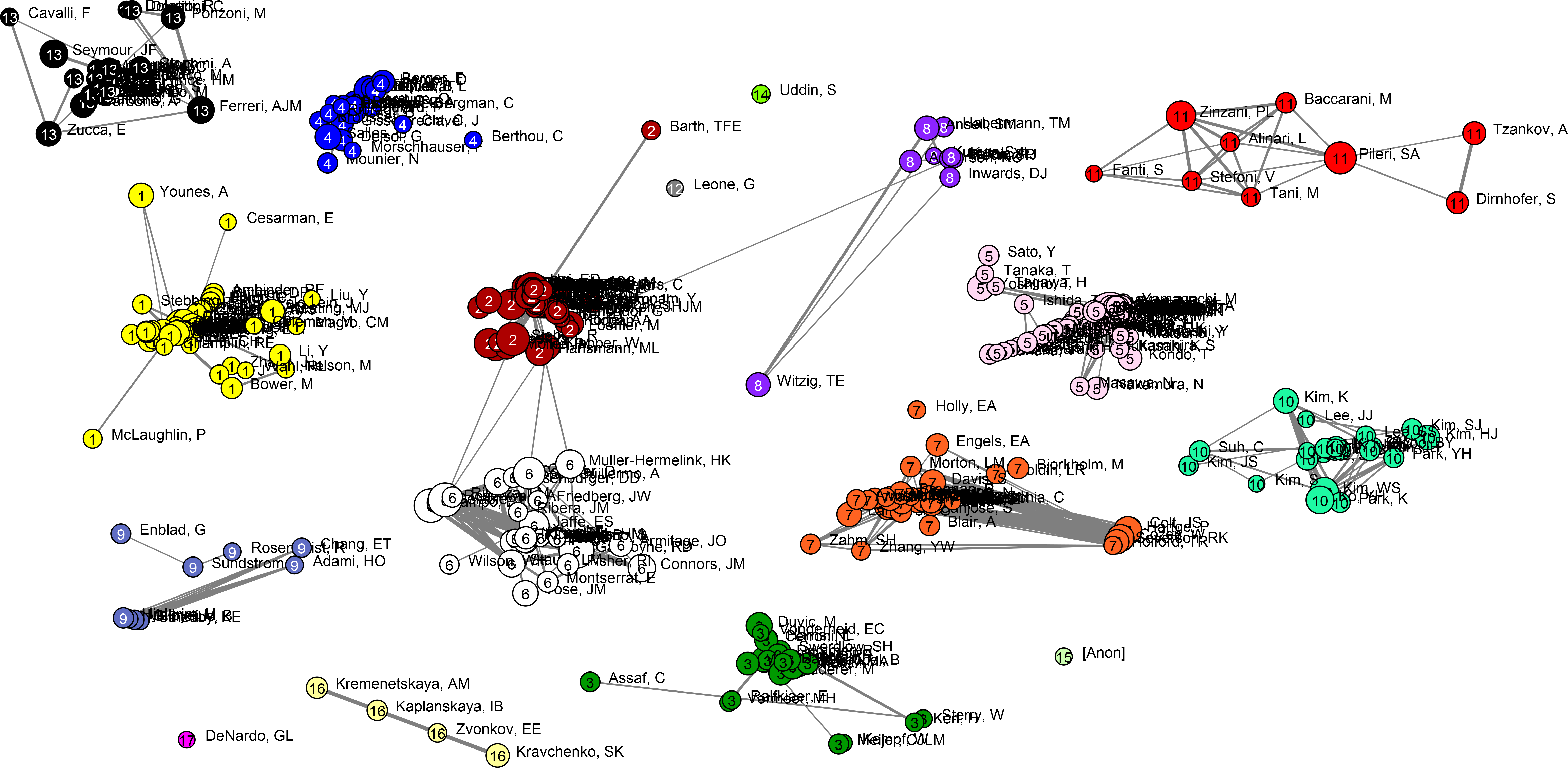

Supplement: S1 Fig — This network maps illustrate the collaboration networks of all authors in the datasets. Each vertex on the map illustrates an author, the size of the vertex indicates the number of papers published by this author, on the respective subjects (S3 Table), in the given time period. The thickness of lines between authors indicates the degree of collaboration between them and the colours indicate which authors are associated with the same cluster. (TIF) [file pone.0116966.s001.tif]

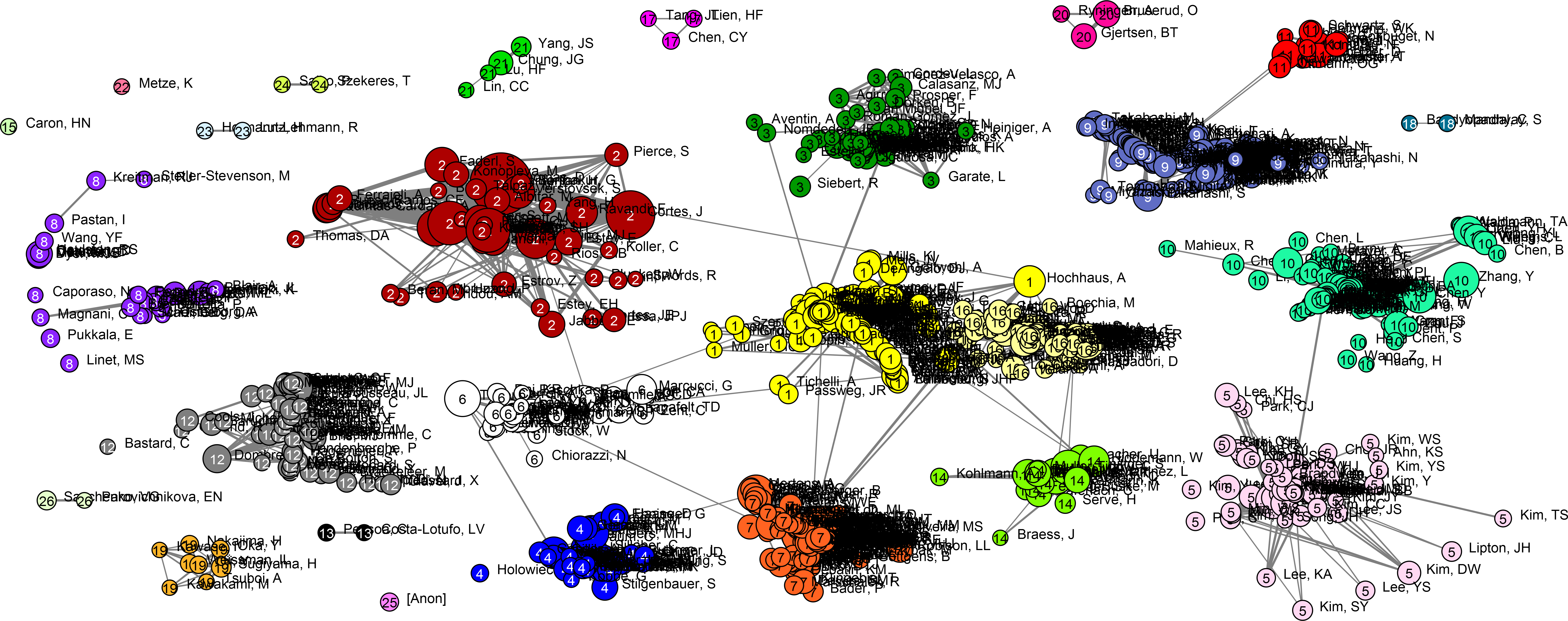

Supplement: S2 Fig — This network maps illustrate the collaboration networks of all authors in the datasets. Each vertex on the map illustrates an author, the size of the vertex indicates the number of papers published by this author, on the respective subjects (S4 Table), in the given time period. The thickness of lines between authors indicates the degree of collaboration between them and the colours indicate which authors are associated with the same cluster. (TIF) [file pone.0116966.s002.tif]

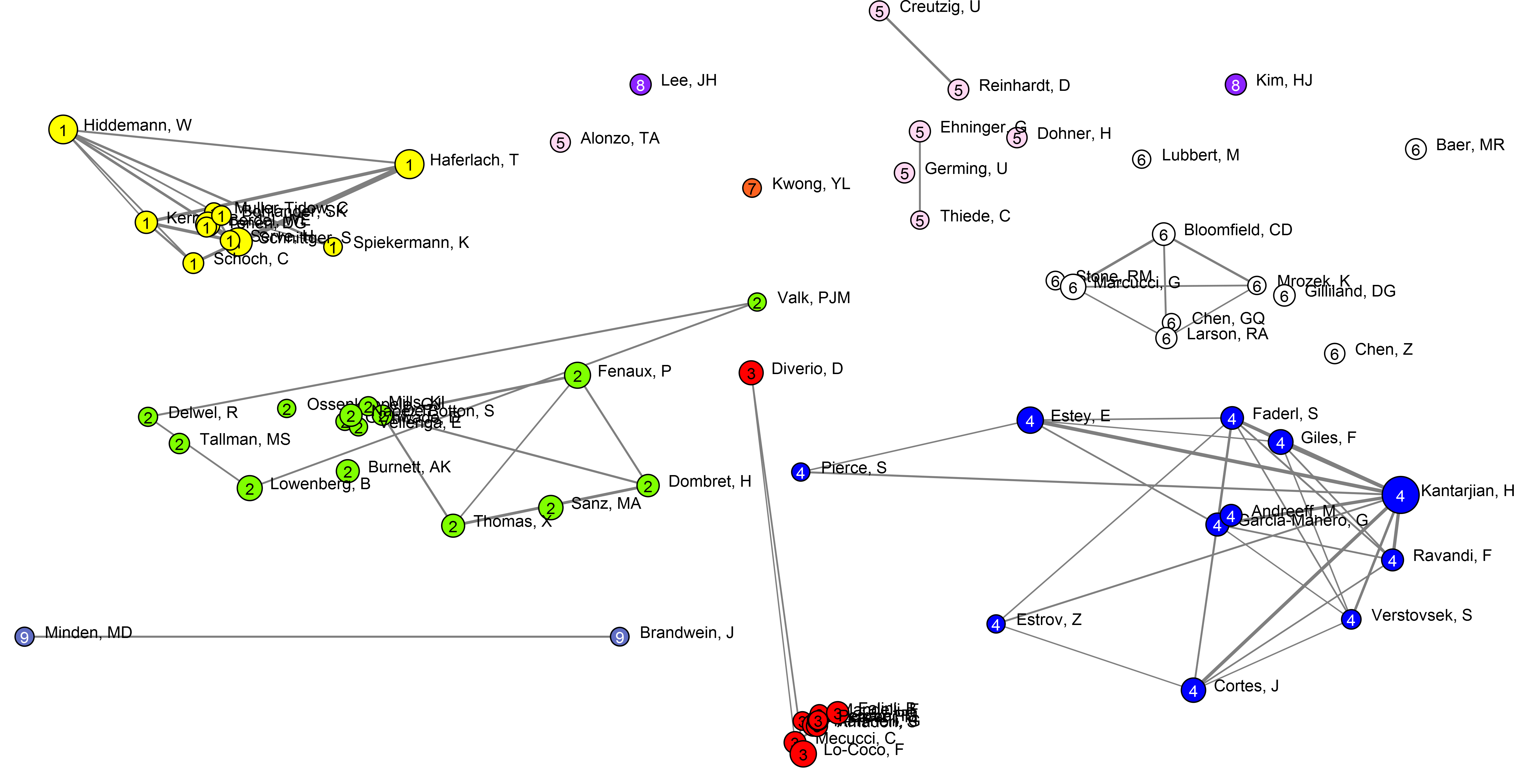

Supplement: S3 Fig — This network maps illustrate the collaboration networks of all authors in the datasets. Each vertex on the map illustrates an author, the size of the vertex indicates the number of papers published by this author, on the respective subjects (S5 Table), in the given time period. The thickness of lines between authors indicates the degree of collaboration between them and the colours indicate which authors are associated with the same cluster. (TIF) [file pone.0116966.s003.tif]
