# Supplementary material for: Global Myeloma Research Clusters, Output, and Citations: A Bibliometric Mapping and Clustering Analysis
Source: PLoS One. 2015 Jan 28;10(1):e0116966. doi: 10.1371/journal.pone.0116966 (PMC4309532; doi:10.1371/journal.pone.0116966)
Supplement: S1 Table — Queries for multiple myeloma, lymphoma, and leukaemia in Science Citation Index-Expanded (SCI-E) and PubMed MEDLINE, delimited to publication years = 2005–2009. The refined SCI-E query contains only articles, reviews, and letters. (PDF) [file pone.0116966.s004.pdf]

Table S1: Search results compared between Web of Science and PubMed

Database	Query	Results (N)
**SCI-E**	"Multiple Myeloma"	11,095
	"lymphoma"	33,138
	"leukaemia" OR "leukemia"	53,515
**Refined SCI-E**	"Multiple Myeloma"	6,970
	"lymphoma"	22,698
	"leukaemia" OR "leukemia"	40,099
**PubMed MEDLINE**	"Multiple Myeloma"[mesh]	5,385
	"lymphoma"[mesh]	19,974
	"leukaemia"[mesh] OR leukaemia OR leukemia	36,719

Queries for Multiple Myeloma (MM), lymphoma, and leukaemia in Science Citation Index-Expanded (SCI-E) and PubMed MEDLINE, delimited to publication years = 2005-2009. The refined SCI-E query contains only articles, reviews, and letters.
