# Supplementary material for: Global Myeloma Research Clusters, Output, and Citations: A Bibliometric Mapping and Clustering Analysis
Source: PLoS One. 2015 Jan 28;10(1):e0116966. doi: 10.1371/journal.pone.0116966 (PMC4309532; doi:10.1371/journal.pone.0116966)
Supplement: S2 Table — Publication counts in SCI-E distributed by publication year. (PDF) [file pone.0116966.s005.pdf]

Table S2: Counts of papers over time

Count	2005	2006	2007	2008	2009
**Multiple Myeloma**	1,144	1,298	1,436	1,459	1,628
**Lymphoma**	4,034	4,240	4,580	4,884	4,960
**Leukaemia**	7,459	7,652	7,964	8,505	8,519

Publication counts in SCI-E distributed by publication year.
