# Supplementary material for: Global Myeloma Research Clusters, Output, and Citations: A Bibliometric Mapping and Clustering Analysis
Source: PLoS One. 2015 Jan 28;10(1):e0116966. doi: 10.1371/journal.pone.0116966 (PMC4309532; doi:10.1371/journal.pone.0116966)
Supplement: S3 Table — Descriptive data for all clusters: Nc = total publication count; pfc = fractional publication count; pfc/Nc = ratio between pfc and Nc, indicating degree of intra-cluster collaboration; µs = mean standardised citation score; PPtop10 = proportion of publications from the top decile; Ccoeff = 1-neighbourhood cluster coefficient; Cclose = mean closeness centrality for the cluster; EU, US, Asia = fraction of addresses on papers from the respective areas. (PDF) [file pone.0116966.s006.pdf]

Table S3 Descriptions of lymphoma research clusters ranked by publication counts.

Cluster ID	N_c	p_{fc}	p_{fc}/ Nc	μ_s	PP_{top10}	C_{coeff}	C_{close}	EU	US	Asia
ly1	1164	298.49	0.256	1.72	0.19	0.456	0.220	15.40%	61.50%	14.90%
ly2	975	253.49	0.26	1.5	0.17	0.686	0.430	76.20%	14.10%	4.50%
ly5	963	215.83	0.224	0.83	0.07	0.527	0.545	10.60%	6.80%	81.10%
ly6	701	160.08	0.228	1.88	0.23	0.884	0.312	57.40%	24.90%	6.10%
ly7	481	151.49	0.315	1.39	0.15	0.868	0.275	59.90%	25.60%	4.40%
ly3	457	116.95	0.256	1.17	0.12	0.787	0.329	67.70%	25.50%	3.60%
ly13	447	115.56	0.259	1.46	0.17	0.618	0.363	76.20%	8.00%	1.20%
ly4	418	102.47	0.245	1.42	0.17	0.817	0.437	86.70%	5.00%	1.20%
ly10	341	86.82	0.255	0.81	0.07	0.750	0.346	1.30%	13.40%	83.30%
ly8	181	40.75	0.225	2.28	0.33	0.827	0.352	21.10%	66.10%	5.20%
ly11	157	41.58	0.265	1.57	0.18	0.919	0.347	84.40%	7.80%	1.70%
ly9	109	29.39	0.27	1.46	0.15	0.893	0.515	77.30%	16.90%	1.20%
ly16	47	13.78	0.293	0.05	0	1	0	4.50%	0.00%	95.50%
ly14	24	2.82	0.117	1.68	0.29	0	0	6.10%	21.20%	0.00%
ly15	21	21	1	0	0	0	0	0%	0%	0%
ly12	20	2.36	0.118	1.06	0.15	0	0	90.50%	4.80%	0.00%
ly17	20	4.13	0.206	0.59	0.05	0	0	13.00%	87.00%	0.00%
