# Supplementary material for: Global Myeloma Research Clusters, Output, and Citations: A Bibliometric Mapping and Clustering Analysis
Source: PLoS One. 2015 Jan 28;10(1):e0116966. doi: 10.1371/journal.pone.0116966 (PMC4309532; doi:10.1371/journal.pone.0116966)
Supplement: S4 Table — Descriptive data for all clusters: Nc = total publication count; pfc = fractional publication count; pfc/Nc = ratio between pfc and Nc, indicating degree of intra-cluster collaboration; µs = mean standardised citation score; PPtop10 = proportion of publications from the top decile; Ccoeff = 1-neighbourhood cluster coefficient; Cclose = mean closeness centrality for the cluster; EU, US, Asia = fraction of addresses on papers from the respective areas. (PDF) [file pone.0116966.s007.pdf]

Table S4 Descriptions of leukaemia research clusters ranked by publication counts.

Cluster ID	N_c	p_{fc}	p_{fc}/Nc	μ_s	PP_{top10}	C_{coeff}	C_{close}	EU	US	Asia
le10	2649	608.9	0.23	1.12	0.12	0.432	0.332	15.60%	35.80%	40.20%
le9	2578	667.09	0.259	0.77	0.07	0.375	0.245	9.60%	11.80%	76.30%
le1	2471	647.82	0.262	1.51	0.18	0.556	0.334	57.70%	27.30%	2.90%
le7	2362	631.5	0.267	1.35	0.15	0.604	0.345	64.70%	22.40%	4.40%
le5	1122	302.57	0.27	1	0.1	0.625	0.452	10.30%	23.30%	55.30%
le16	1098	325.12	0.296	1.41	0.16	0.640	0.320	85.80%	8.70%	0.90%
le2	1007	435.37	0.432	1.97	0.25	0.782	0.466	19.50%	67.80%	6.10%
le4	969	270.41	0.279	2.11	0.25	0.653	0.441	72.20%	20.50%	4.40%
le6	851	273.78	0.322	2.49	0.28	0.680	0.345	25.40%	60.60%	5.80%
le12	848	204.34	0.241	1.51	0.16	0.648	0.324	86.30%	7.30%	1.50%
le3	762	175.71	0.231	1.29	0.15	0.673	0.296	87.00%	8.50%	0.90%
le8	519	140.54	0.271	1.32	0.16	0.731	0.335	70.30%	20.40%	3.30%
le14	351	131.03	0.373	1.37	0.18	0.811	0.468	80.20%	10.60%	2.80%
le11	305	74.96	0.246	1.35	0.15	0.829	0.471	47.00%	24.80%	24.40%
le19	84	21.59	0.257	2.13	0.25	0.940	0.146	12.30%	30.20%	55.70%
le21	65	15.39	0.237	0.79	0.03	1	0	0.00%	9.90%	90.10%
le20	58	22.25	0.384	0.77	0.05	1	0	94.50%	2.70%	1.40%

le17	39	8.61	0.221	0.81	0.1	1	0	2.30%	13.60%	84.10%
le26	31	6.79	0.219	0.02	0	0	0	0.00%	0.00%	100.00%
le25	27	27	1	0	0	0	0	0%	0%	0%
le18	25	6.38	0.255	0.55	0	0	0	13.30%	23.30%	63.30%
le23	25	5.63	0.225	0.86	0.08	0	0	89.70%	5.10%	0.00%
le13	24	4.74	0.198	0.51	0	0	0	4.00%	0.00%	0.00%
le24	23	4.34	0.189	1	0.04	0	0	77.30%	9.10%	2.30%
le22	21	7.45	0.355	0.46	0	0	0	9.10%	0.00%	0.00%
le15	19	3.73	0.196	1.03	0.05	0	0	90.50%	0.00%	0.00%
