# Supplementary material for: Global Myeloma Research Clusters, Output, and Citations: A Bibliometric Mapping and Clustering Analysis
Source: PLoS One. 2015 Jan 28;10(1):e0116966. doi: 10.1371/journal.pone.0116966 (PMC4309532; doi:10.1371/journal.pone.0116966)
Supplement: S5 Table — Descriptive data for all clusters: Nc = total publication count; pfc = fractional publication count; pfc/Nc = ratio between pfc and Nc, indicating degree of intra-cluster collaboration; µs = mean standardised citation score; PPtop10 = proportion of publications from the top decile; Ccoeff = 1-neighbourhood cluster coefficient; Cclose = mean closeness centrality for the cluster; EU, US, Asia = fraction of addresses on papers from the respective areas. (PDF) [file pone.0116966.s008.pdf]

Table S5 Descriptions of acute myeloid leukaemia research clusters ranked by publication counts.

Cluster ID	N_c	p_{fc}	p_{fc}/Nc	μ_s	PP_{top10}	C_{coeff}	C_{close}	EU	US	Asia
am2	243	47.47	0.195	1.47	0.17	0.788	0.361	75.90%	11.40%	7.60%
am6	171	30.78	0.18	2.17	0.31	0.778	0.448	29.60%	52.10%	15.60%
am3	151	22.51	0.149	1.46	0.19	0.864	0.449	89.60%	7.50%	0.70%
am1	147	42.73	0.291	1.6	0.22	0.861	0.301	75.30%	16.70%	3.50%
am4	126	40.91	0.325	2.13	0.3	0.952	0.137	20.80%	70.80%	3.40%
am5	120	18.28	0.152	1.52	0.22	0.786	0.287	71.70%	19.40%	2.30%
am8	45	5.59	0.124	0.23	0	0	0	0.00%	4.30%	95.70%
am9	27	5.45	0.202	0.98	0.15	0	0	30.80%	13.50%	1.90%
am7	20	4.37	0.219	0.53	0.05	0	0	0.00%	0.00%	95.20%
